# Supplementary material for: PPIscreenML: Structure-based screening for protein-protein interactions using AlphaFold
Source: bioRxiv. 2024 Apr 30:2024.03.16.585347. Originally published 2024 Mar 17. Preprint. [Version 2] doi: 10.1101/2024.03.16.585347 (PMC10979958; doi:10.1101/2024.03.16.585347)
Supplement: 1 [file NIHPP2024.03.16.585347V2-supplement-1.pdf]

bioRxiv preprint doi: https://doi.org/10.1101/2024.03.16.585347; this version posted April 30, 2024. The copyright holder for this preprint (which was not certified by peer review) is the author/funder, who has granted bioRxiv a license to display the preprint in perpetuity. It is made available under aCC-BY 4.0 International license.

Supplemental Figures

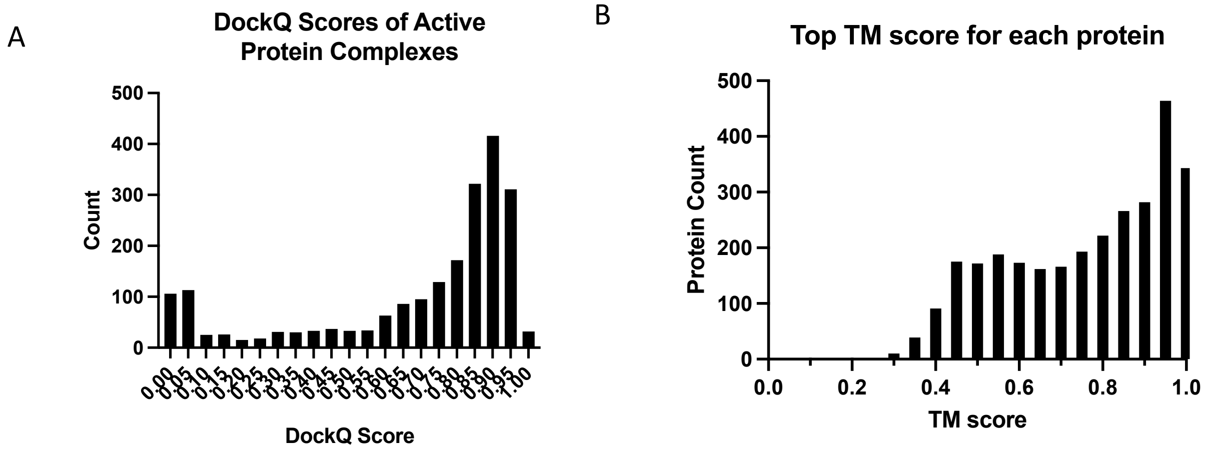

Figure S1: Composition of the active and decoy datasets. (A) For the set of actives, the distribution of DockQ scores are shown (calculated relative to the PDB structure for each complex). Complexes are included in the dataset only if their DockQ scores are at least 0.23, but most DockQ scores for these active pairings are much higher. **(B)** Compelling decoy complexes are assembled by drawing an alternate protein with high TM score relative to the components of the parental (active) complex. In most cases decoy complexes are assembled using component proteins with TM-scores of at least 0.5, corresponding to close structural matches.

bioRxiv preprint doi: https://doi.org/10.1101/2024.03.16.585347; this version posted April 30, 2024. The copyright holder for this preprint (which was not certified by peer review) is the author/funder, who has granted bioRxiv a license to display the preprint in perpetuity. It is made available under aCC-BY 4.0 International license.

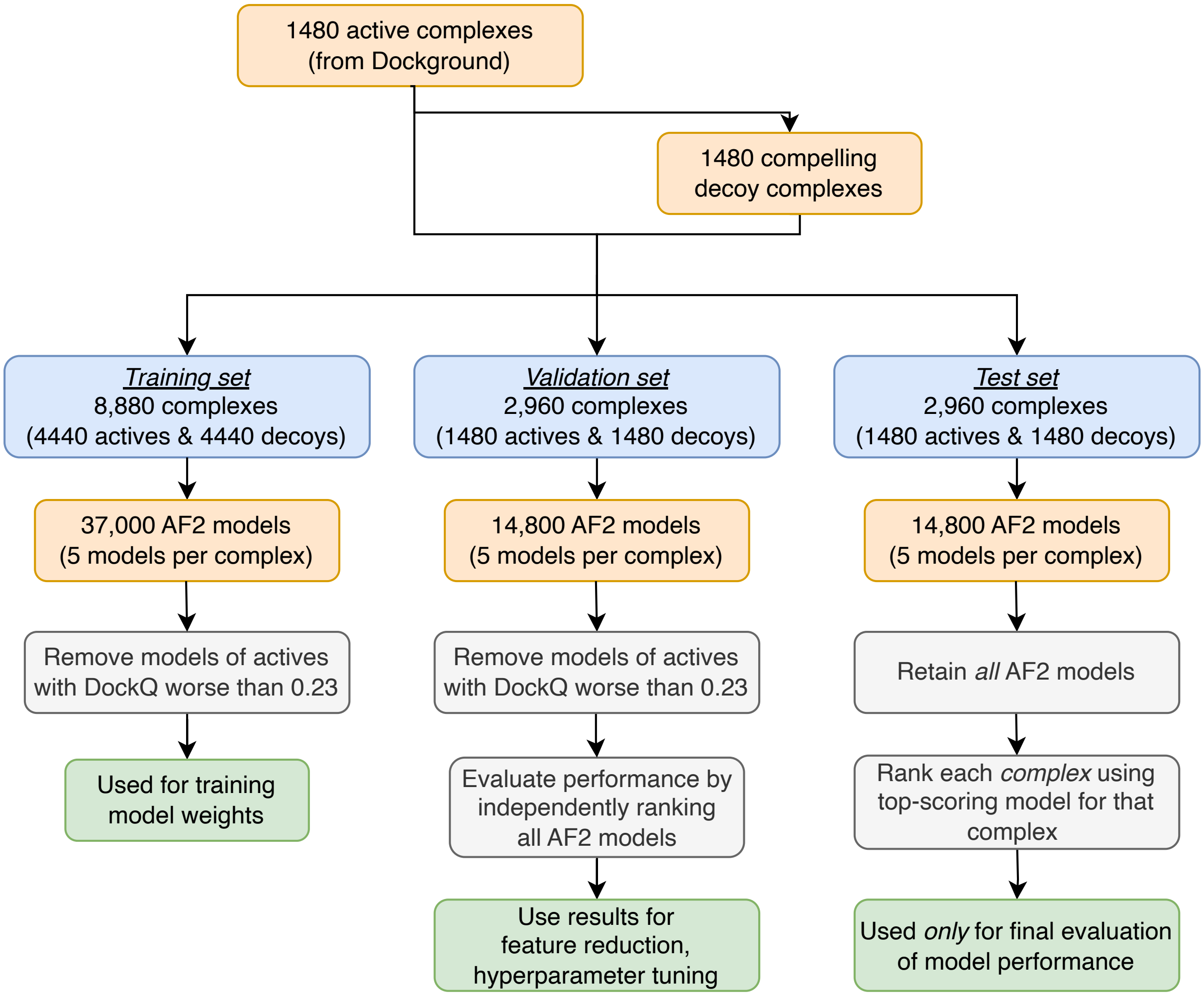

Figure S2: Overview of dataset construction and evaluation. Each active complex was used to generate one compelling decoy complex. Complexes were divided into training / validation / test sets, and 5 AF2 models were built for each complex. Key differences in how the sets were constructed / evaluated are highlighted in *white*. First, the AF2 models of active complexes in the training and validation sets were filtered to keep only correctly-docked models (DockQ>0.23); by contrast, *all* models were retained in the test set with no filtering. Second, performance of the validation set was evaluated by ranking all AF2 models independently; by contrast, performance of the test set was evaluated by ranking each *complex*, using the best-scoring AF2 model for each complex.

bioRxiv preprint doi: https://doi.org/10.1101/2024.03.16.585347; this version posted April 30, 2024. The copyright holder for this preprint (which was not certified by peer review) is the author/funder, who has granted bioRxiv a license to display the preprint in perpetuity. It is made available under aCC-BY 4.0 International license.

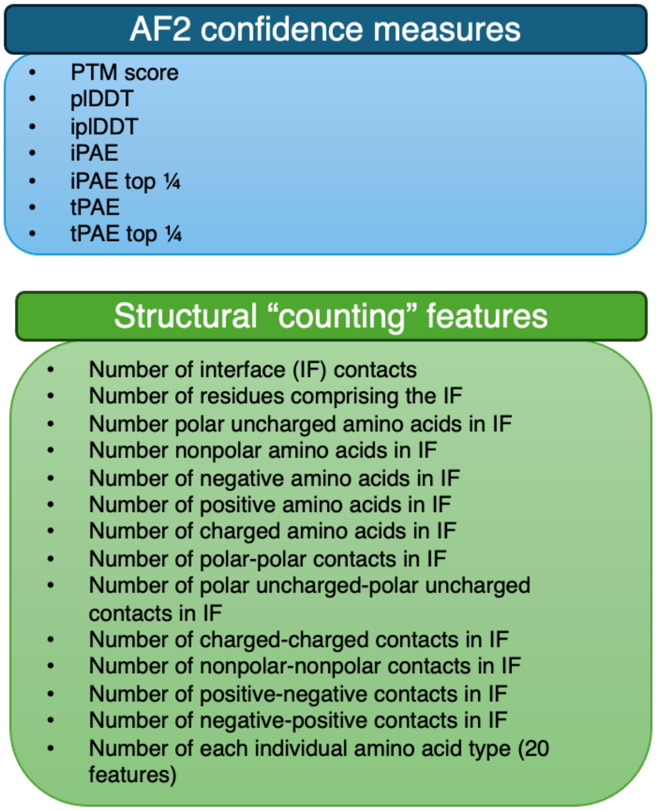

Figure S3: List of 57 total features considered when training PPIscreenML. A list of features extracted from the structural models are shown and grouped into three categories: features from AF2 confidence measures, structural "counting" features extracted using the python package Biopandas, and features from the Rosetta energy function.

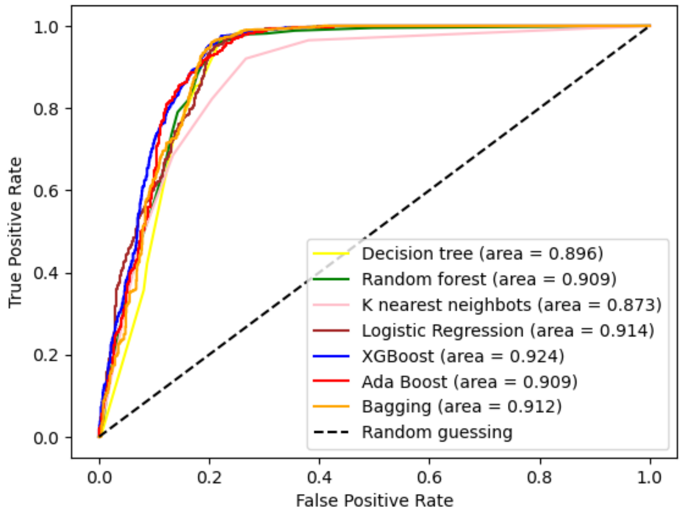

Figure S4: Comparisons of different machine learning classifiers. ROC plots for classifiers built using different machine learning frameworks (each uses all 57 features).

bioRxiv preprint doi: https://doi.org/10.1101/2024.03.16.585347; this version posted April 30, 2024. The copyright holder for this preprint (which was not certified by peer review) is the author/funder, who has granted bioRxiv a license to display the preprint in perpetuity. It is made available under aCC-BY 4.0 International license.

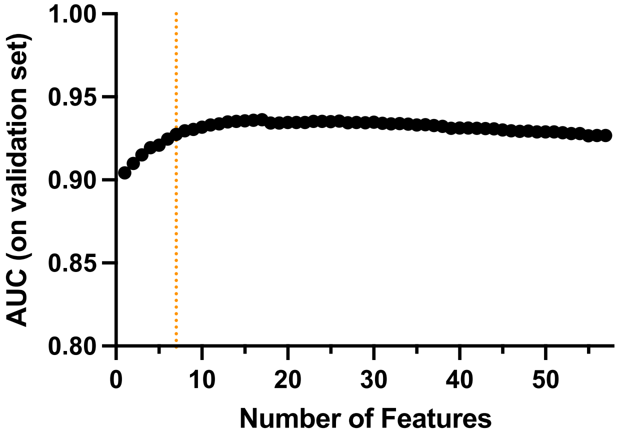

Figure S5: Feature reduction for PPIscreenML. Sequential backwards selection was used to characterize models with diminishing numbers of features. Performance of each candidate model was evaluated on the validation set (drawn from the training set). The vertical dashed line indicates the model selected for PPIscreenML (7 features).

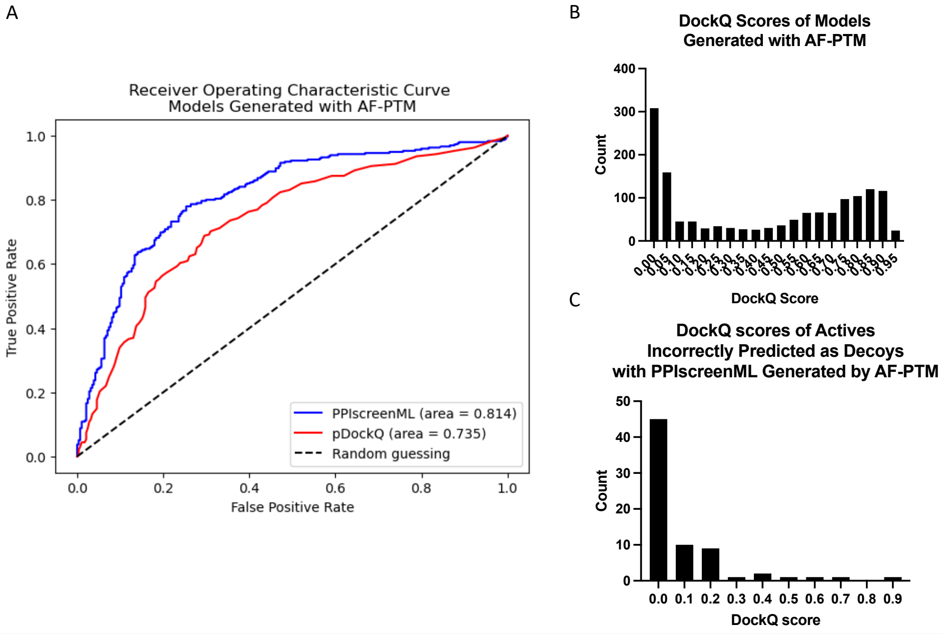

Figure S6: PPIscreenML performance on models generated with AF-ptm. (A) ROC curve of PPIscreenML and pDockQ tested on models built with AF-ptm. iPTM is not included because it is not available for this version of AF. **(B)** DockQ distribution for actives in the test set. **(C)** Among actives that were incorrectly classified by PPIscreenML as “not interacting”, most were mis-docked by AF-ptm.

bioRxiv preprint doi: https://doi.org/10.1101/2024.03.16.585347; this version posted April 30, 2024. The copyright holder for this preprint (which was not certified by peer review) is the author/funder, who has granted bioRxiv a license to display the preprint in perpetuity. It is made available under aCC-BY 4.0 International license.

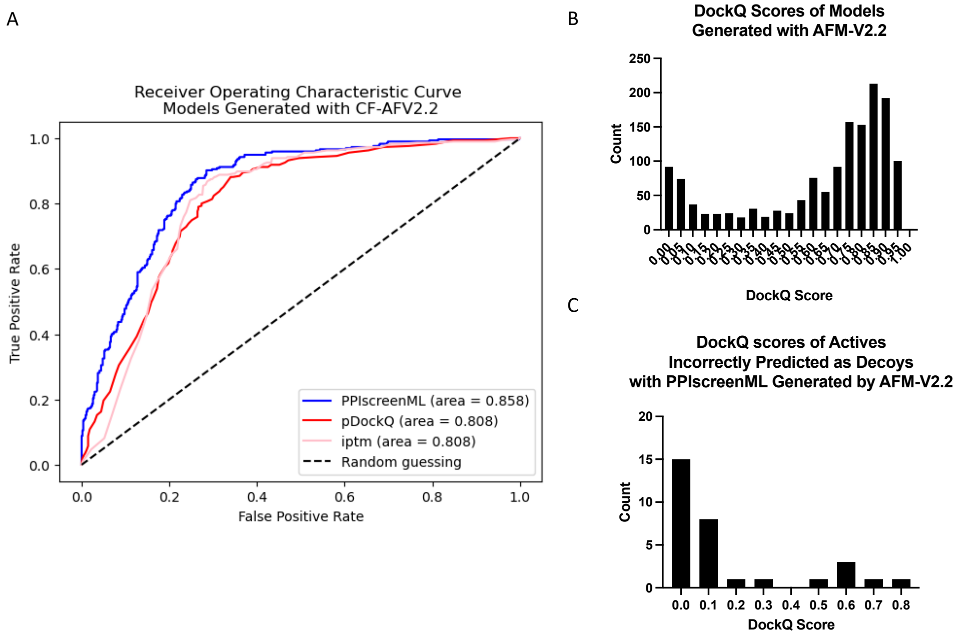

Figure S7: PPIscreenML performance on models generated with AF-multimer version 2.2. **(A)** ROC curve of PPIscreenML and pDockQ tested on models built with **AF-multimer-2.2**. **(B)** DockQ distribution for actives in the test set. **(C)** Among actives that were incorrectly classified by PPIscreenML as "not interacting", most were mis-docked by AF-multimer-2.2.

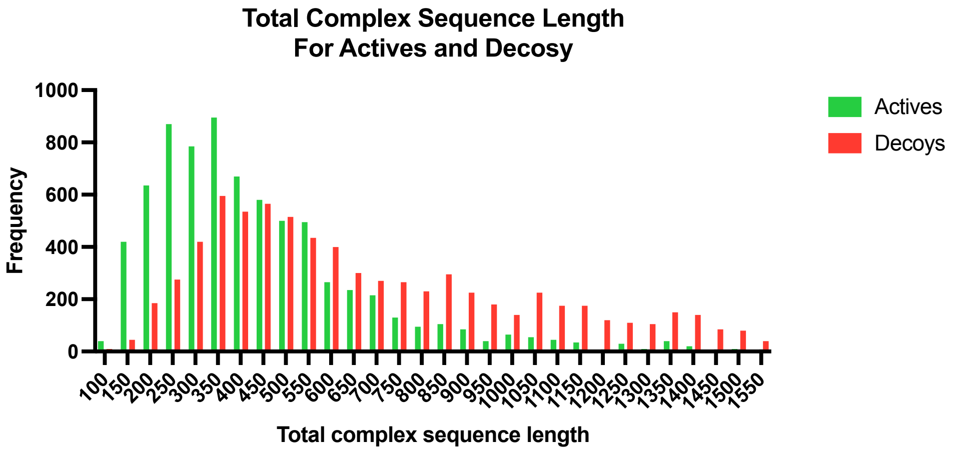

Figure S8: Overlaid histogram of total sequence length between actives and decoys. The method we describe for building compelling decoy complex does exhibit a slight bias for building decoys pairs from component proteins larger than the starting template. This arises from the use of TM-align to define structural analogs, because large query proteins are slightly more likely to yield high scores than small query proteins (for a given template protein). This artifact can allow a model to "cheat" if it includes any features that can serve as a proxy for the total number of residues in a model; accordingly, we ensured that no such features were included in developing PPIscreenML. Importantly, this artifact does not affect the composition or structural features of the generated decoy complexes, which are not systematically different from the active complexes.
